# Clinicopathological analysis of 22 Müllerian adenosarcomas and the sequencing of DICER1 mutation

**DOI:** 10.1186/s13000-024-01477-2

**Published:** 2024-04-03

**Authors:** Xiaohong Yao, Wei Wang, Ying He

**Affiliations:** 1 Department of Pathology, West China Second University Hospital, Sichuan University, Chengdu, 610041 China; 2 Key Laboratory of Birth Defects and Related Diseases of Women and Children (Sichuan University), Ministry of Education, Chengdu, 610041 China

**Keywords:** Adenosarcoma, Genetic screening, Hotspot mutations, DICER1 mutation

## Abstract

**Background:**

Müllerian adenosarcoma, a rare malignancy, presents diagnostic and therapeutic challenges. In this study, we conducted an analysis of the clinicopathological characteristics of 22 adenosarcomas, with a particular focus on screening for DICER1 hot mutations.

**Methods:**

The cohort consisted of patients with adenosarcoma who were registered at the West China Second Hospital between the years 2020 and June 2022. Sanger sequencing was employed to screen for somatic Hotspot mutations in the RNase IIIb domain of DICER1 in the 22 adenosarcomas.

**Results:**

Only one patient exhibited a DICER1 mutation that was not a DICER1 Hotspot mutation. Among the 22 patients, all underwent total hysterectomy with bilateral salpingo-oophorectomy, and 14 out of these 22 patients received adjuvant treatment.

**Conclusion:**

In summary, our study of 22 Müllerian adenosarcomas focused on the clinicopathological features and the presence of DICER1 Hotspot mutations. Although our findings did not reveal any DICER1 mutations in the studied samples, this negative result provides valuable information for the field by narrowing down the genetic landscape of adenosarcomas and highlighting the need for further research into alternative molecular pathways driving this malignancy.

## Background

Adenosarcomas, although infrequent, are not classified as rare mixed tumors of the female genital tract, occupying an intermediate position between adenofibroma and carcinosarcoma [1, 2]. The term Müllerian adenosarcoma was first introduced in 1974 and is characterized by the presence of both benign neoplastic glands and sarcomatous stroma. Tumors display phyllodes-like architecture and the presence of dense cellularity surrounding the glands, leading to the formation of “periglandular cellular cuffs.” [3] Despite several studies investigating molecular genetic profiling of adenosarcomas, specific molecular markers for their diagnosis are currently lacking. It is worth noting that DICER1 plays a vital role in the postnatal differentiation of Müllerian duct mesenchyme derived tissues in the female reproductive tract and is crucial in the biogenesis of microRNAs, which are significant regulators of female reproductive tract development and fertility [4–8]. Mutations in DICER1 have been observed in various sarcomas, with a particular prevalence in the genitourinary tract. Adenosarcomas have been found to exhibit DICER1 mutations in approximately 20% of cases, primarily as somatic mutations [9–13]. The objective of this study was to examine the clinical, pathological, and immunohistochemical characteristics of twenty-two cases of adenosarcoma. Additionally, Sanger sequencing was used to assess the presence of DICER1 RNase IIIb domain mutations in exons 24 and 25 in all 22 tumors.

## Methods

### Case selection

A total of twenty-two adenosarcomas, diagnosed between the period of 2020 and June 2022, were obtained from the West China the second Hospital. The original surgical slides as well as subsequent tumor samples were thoroughly examined. Each tumor was evaluated for the occurrence of stromal overgrowth, the presence of heterologous differentiation, and the grade of the mesenchymal component. The diagnosis of stromal sarcomatous overgrowth was established when the sarcomatous component of the tumor was estimated to encompass a minimum of 25% of the tumor volume [14]. A comprehensive analysis was performed to gather clinical and pathologic data pertaining to patients’ age, presentation, tumor size, gross features, stage, immunohistochemistry, treatment, and telephone follow-up.

### DICER1 sequencing

Formalin-fixed, paraffin-embedded (FFPE) tumor tissue with the highest tumor content was obtained from all 22 adenosarcomas. In order to conduct this analysis, Formalin-fixed, paraffin-embedded (FFPE) tumor tissue samples with the highest tumor content were procured from all 22 adenosarcomas. Subsequently, PCR amplification followed by Sanger sequencing was employed to screen the DNA sequences encompassing the RNase IIIb Hotspot mutation sites in the 22 tumors.

## Results

The result of the clinical, pathological and immunohistochemical features of the 22 adenosarcomas are presented in Table 1.

**Table 1:** Clinical and pathological features of Mullerian adenosarcomas

case	Age(y)	Size(cm)	Site	Depth of myometrial invasion	Extreuterine disease	Stage	LVI	SO	Grade	HET	Treatment	Follow-up(mouth)	CD10	ER	PR	Desmin	ki67	Caldesmon	CyclinD1+	DICER1
case1	52	8.5	Uterine	100%	rectum	IVa	-	-	low	-	TH + BSO,CT	20,NED	+	+	+	+	10%-15%	+	-	-
case2	47	13.6	Uterine	-	left broad ligament, right fallopian tube mesanga, rectum, rectovaginal pouch, omentum	IVa	-	+	low	-	TH + BSO,CT	20,NED	+	...	...	-	40%	-	...	-
case3	36	7.5	Uterine	< 50%	-	Ib	-	+	low	-	TH + BSO	29,NED	+	+	+	+	5%-10%	+	-	-
case4	32	2.7	Uterine	-	-	Ia	-	-	low	-	PE + TH + BSO,ET	29,NED	+	...	...	+	10%	+	...	-
case5	45	9.5		-	-	Ia	-	-	low	-	TH + BSO	...	+	+	+	+	15%	-	+	-
case6	46	2	Cervix	< 50%	-	Ib	-	-	low	-	PE + TH + BSO	26,NED	+	+	+	+	< 5%	-	-	-
case7	52	3.8	Uterine	< 50%	mesostenium, transvers mesocolon	IIIb	-	-	low, undifferen-tiated	Rh	PE + TH + BSO,CT	21,NED	+	+	+	+	60%	...	...	-
case8	47	4	Cervix	< 50%	-	Ib	-	-	low	SCLE	PE + TH + BSO,CT	4,NED	+	+	+	+	5%	-	-	-
case9	42	7	Cervix	< 50%	pelvic lymphnodes	IIIC	+	-	high	-	TH + BSO,NCT + CT	...	-/+	-/+	-/+	-/-	-/<80%	-	-/+	-
case10	62	3.5	Uterine	100%	bilateralovary, omentum, mesostenium, rectum, bladder, sacral ligament	IVa	-	+	high	-	TH + BSO,NCT + CT	14,NED	+	+	-	+	5%	+	-	-
case11	25	7.5	Uterine	< 50%	-	Ib	+	+	undifferen-tiated	-	TH + BSO,CT	12,NED	-	-	-	-	40%	-	-	-
case12	51	...	Uterine	-	-	Ia	-	-	low	-	PE + TH + BSO,CT	30,NED	-/+	...	...	...	5%/15%	...	...	-
case13	42	10	Left ovary	-	-	Ia	-	-	low	-	TH + USO	...	+	+	+	+	25%	-	+	-
case14	44	6	Uterine	> 50%	-	Ic	-	-	low	-	TH + BSO,CT	14,NED	+	...	...	+	20%	...	...	-
case15	51	2	Uterine	-	-	Ia	-	-	low	-	TH + BSO	...	+	-	-	-	20%	-	+	-
case16	53	6	Uterine	100%	-	Ic	-	-	low	-	TH + BSO,CT	36,NED	+	+	+	+	30%	-	-	+
case17	52	8.5	Uterine	> 50%	lymphnodes	IIIc	+	+	undifferen -tiated	-	TH + BSO	...	-	-	-	-	40%	-	-	-
case18	39	1.7	Uterine	100%	-	Ic	-	-	low	-	PE + TH + BSO,CT	17,NED	-	-	+	-	25%	-	-	-
case19	54	1.5	Uterine	< 50%	-	Ib	-	+	low	-	TH + BSO	...	+	+	+	-	70%	-	-	-
case20	29	4	Uterine	> 50%	-	Ic	-	-	low	-	PE + TH + BSO, CT	15 NED	-/++	++/++	+++/+	...	5%-10%	-/-	-/-	-
case21	26	12.5	Uterine	> 50%	-	Ic	-	-	low	-	PE + TH + BSO, ET	15 NED	-/++	+++/+++	+++/+++	...	5%/15%	-/+	-/-	-
case22	42	1.8	Uterine	> 50%	-	Ic	-	-	low	-	TH + BSO	12 NED	-	-	-	...	10%	...	+	-

+=present, -=absent; LVI: lymphnodes vascular invasion; SO: sarcomatous overgrowth; HET: heterologous conponent; Rh: rhabdomyosarcoma; SCLE: sex cordlike elements; TH + BSO: Total hysterectomy bilateral salpingo-oophorectomy; USO unilateral salpingo-oophorectomy; PE: polypectomy; CT: chemotherapy; NCT: neoadjuvant chemotherapy; ET endocrinotherapy; NED: no evidence of disease

### Clinical features

The age range of the patients at the time of diagnosis was 25 to 62 years, with a median age of 45.5. Among the patients, 21 had uterine tumors, with 18 located in the corpus and 3 in the cervix. Only one patient had an ovarian tumor. Additionally, 6 patients had metastases at the time of diagnosis, with one case localized in the rectum and the other exhibiting extensive metastases in the abdominopelvic cavity. The presenting symptoms included irregular vaginal bleeding in 9 out of 22 cases, abdominal discomfort in 10 out of 22 cases, and polypoid or pelvic mass in 7 out of 22 cases. For ovarian adenosarcoma, the primary presenting symptom is a pelvic mass. The initial surgical procedures performed included total hysterectomy/salpingo-oophorectomy in 14 out of 22 patients, and polypectomy in the remaining 8 out of 22 patients. Subsequently, the latter eight patients also underwent total hysterectomy/salpingo-oophorectomy. Additionally, two patients received endocrinotherapy, while 12 patients received chemotherapy. Among the latter two patients, neoadjuvant chemotherapy was also administered. The staging of uterine sarcoma according to the 2015 International Federation of Gynecology and Obstetrics (FIGO) guidelines, as well as the staging of ovarian carcinoma according to the 2017 FIGO guidelines, were as follows: out of the 21 uterine tumors, 4 were classified as stage 1 A, 5 as stage IB, 6 as stage IC, 1 as stage IIIB, 2 as stage IIIc, and 3 as stage IVa.

### Pathological features

**Fig. 1:**
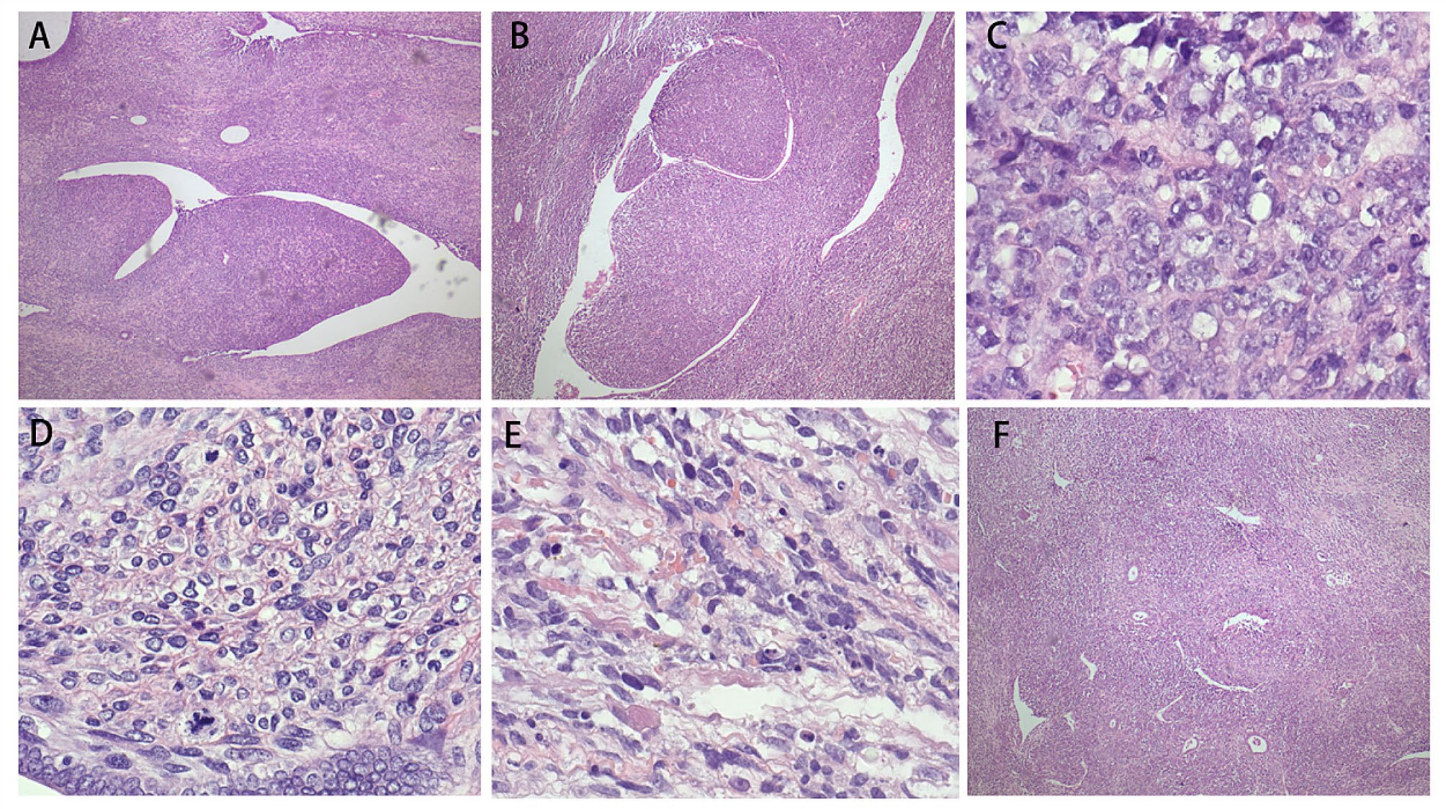
Representative haematoxylin and eosin-stained images. (**A**)(**B**) Phyllodes-like structure are composed of benign epithelium and mesenchymal lesion. (**C**) High-grade sarcomatous component. (**D**) Mitotic index of the subepithelial stroma. (**E**) Rhabomyosarcomatous differentiation. (**F**) Sacomatous overgrowth

Five patients were accurately diagnosed preoperatively through histologic examinations of resected lesions, while the remaining patients received a diagnosis through hysterectomy. Among the patients, seven presented with polypoid or nodular masses, typically protruding into the endometrial cavity, endocervix, or ovary. The mean tumor size was 5.9 cm (range: 1.5 to 13.6 cm). In case 14 and case 17, the tumors exhibited both solid and cystic components. The solid areas appeared yellow-white, while the cysts contained either mucoid material or hemorrhagic fluid. Additionally, two tumors displayed grossly evident focal hemorrhage and necrosis. Microscopic examination revealed the presence of a combination of benign neoplastic glands and sarcomatous stroma. The distribution of the glands was generally uniform, except for areas of sarcomatous overgrowth observed in six out of twenty-two cases. The epithelial component, typically endometrioid in nature, occasionally exhibited eosinophilic and squamous epithelium, as seen in case twelve, resembling the proliferative endometrium. The stromal component, predominantly of low grade (eighteen out of twenty-two cases), consisted of round, spindle-shaped nuclei, limited eosinophilic cytoplasm, and unclear cell boundaries. Periglandular cuffs with heightened cellularity were observed in all cases. The stromal cells within these periglandular cuffs exhibited greater cellular atypia and increased mitotic activity compared to the surrounding hyaline or fibrous areas. Within the stromal component, nuclear atypia ranged from mild to moderate, and the mitotic count varied from less than 1 to 28 per 10 high-power fields. Among the six tumors with sarcomatous overgrowth, two had undifferentiated stromal components, one patient had a high-grade tumor, and the remaining three tumors displayed only moderate nuclear atypia. Case 7 exhibited embryonal rhabdomyosarcoma differentiation, while case 8 demonstrated sex cordlike differentiation (Fig. 1). The adenosarcomas located in the left ovary were characterized as low-grade sarcomas and were found to be associated with endometriosis.

### Immunohistochemical stains and genetic features

Notably, there is a lack of immunohistochemical markers that can definitively diagnose adenosarcoma, thus rendering the diagnosis heavily reliant on morphologic features. Among the sarcomatous component of adenosarcomas, the most frequently observed immunohistochemical markers include CD10, which tested positive in 18 out of 22 cases. Additionally, other potential markers that may be present are estrogen receptor (ER) in 13 out of 18 cases, progesterone receptor (PR) in 13 out of 18 cases, and desmin in 11 out of 18 cases. Additional markers that may be present include estrogen receptor (ER) 13/18, progesterone receptor (PR) 13/18, and desmin 11/18.

Sanger sequencing was performed on all 22 patients to determine the presence of DICER1 hot mutations. The results revealed that only one patient (case16) exhibited the c.5416_5417insA mutation. Interestingly, no DICER1 mutation was observed in cases7, which exhibited embryonal rhabdomyosarcoma differentiation.

## Discussion

DICER1, a highly conserved ribonuclease, is essential for embryonic development as demonstrated by the embryonic lethality observed in dicer1 homozygous knockout mice [4, 5]. The Dicer protein, a member of the ribonuclease (RNase) III family, plays a crucial role in the production of small RNAs such as microRNAs and small interfering RNAs, which act as negative regulators of gene expression [6, 15]. Pathogenic somatic mutations primarily occur as missense substitutions in specific regions of the RNase IIIb domain, specifically exons 24 and 25, affecting conserved metal-ion-binding residues (E1705, D1709, G1809, D1810, and E1813) [16]. In our study, we conducted screening for RNase IIIb Hotspot mutation sites in adenosarcoma tumors and found no DICER1 mutations. This result can be attributed to the rarity of DICER1 mutations in adenosarcoma and the limited sample size of only 22 tumors tested. However, a review of 79 gynecological sarcomas has reported the presence of at least one DICER1 mutation, either somatic or germline. The most prevalent tumor types with DICER1 mutations were embryonal rhabdomyosarcoma (ERMS) (*n* = 47, 59%) and adenosarcoma (*n* = 22, 28%) [14]. Furthermore, in a targeted genomic analysis of adenosarcoma, two out of 18 tumors were found to have DICER1 mutations [17]. The presence of DICER1 mutation was observed in 2 out of 9 cases of high-grade adenosarcoma, while no mutation was detected in low-grade adenosarcoma [18]. Piscuoglio et al. also identified DICER1 mutation in 2 out of 19 cases with rhabdomyosarcoma differentiation [19]. However, it should be noted that DICER1 mutations have been primarily reported in a small subset of adenosarcomas with rhabdomyosarcomatous elements, but this alteration is not exclusively associated with heterologous rhabdomyosarcomatous differentiation [11]. Furthermore, there is a case report documenting the presence of germline DICER1 mutation in adenosarcoma [7]. 

On the other hand, the utilization of FFPE tissue samples in research poses various challenges. The process of fixation and embedding has a significant impact on molecular biology assays as it leads to fragmentation and chemical alterations of nucleic acids. Moreover, the extraction of DNA from FFPE tissue is often a complex endeavor. Additionally, when analyzing a limited tumor region amidst normal tissue, the presence of mutations may be diluted and potentially overlooked.

In terms of medical practice, the recommended treatment approach typically involves total hysterectomy with bilateral salpingo-oophorectomy, as lymph node metastasis is infrequent. The role of adjuvant radiotherapy and chemotherapy remains uncertain and requires further comprehensive evaluation. In our study, a total of 22 patients underwent total hysterectomy with bilateral salpingo-oophorectomy, and among these patients, 14 out of 22 received adjuvant treatment, which included chemotherapy, endocrinotherapy, and neoadjuvant chemotherapy. Factors such as deep myometrial invasion, sarcomatous overgrowth, heterologous elements, lymphovascular space invasion (LVSI), and advanced stage have all been associated with a poorer prognosis [20, 21]. However, the extent of influence exerted by these factors exhibits variability, and at present, there exists no conclusive evidence pertaining to the comparative significance of these risk factors in terms of prognosis. Nevertheless, Michael J. Nathenson et al. have identified age, presence of sarcomatous overgrowth, presence of myometrial invasion, presence of lymphovascular invasion, and lymph node involvement as the most crucial prognostic factors for uterine adenosarcoma. It is worth noting that conservative management of adenosarcoma is infrequent, and all women who underwent uterine-preserving surgery were classified as FIGO stage I [22]. It is worth noting that conservative management of adenosarcoma is infrequent, and all women who underwent uterine-preserving surgery were classified as FIGO stage I [22–26]. Given the increasing global trend of delayed motherhood and the earlier diagnosis of adenosarcoma, there is a foreseeable rise in the demand for fertility preservation. In certain instances of early-stage adenosarcoma, fertility-sparing treatments may be utilized. However, due to the risk of recurrence, it is imperative to conduct thorough post-operative monitoring and follow-up. Once the woman has completed her desired family size, completion surgery becomes necessary [27]. Due to the favorable overall prognosis associated with these tumors, further definitive data is required to determine the potential benefits of overtreatment and adjuvant therapy for low-risk patients.

## Conclusion

In conclusion, our study provides a detailed examination of the clinical, pathological, and genetic characteristics of Müllerian adenosarcomas, with a specific focus on DICER1 Hotspot mutations. While we did not identify any DICER1 Hotspot mutations in our cohort, these findings contribute to our understanding of the genetic heterogeneity of adenosarcomas. Moving forward, it will be essential to explore additional molecular pathways and genetic alterations that may contribute to the pathogenesis of adenosarcomas. These insights will be crucial for the development of more targeted and effective treatment strategies for patients with this rare malignancy.

## Data Availability

Not applicable.
